# The Most Effective Methods for Delivering Severe Weather Early Warnings to Fishermen on Lake Victoria

**DOI:** 10.1371/currents.dis.d645f658cf20bc4a23499be913f1cbe1

**Published:** 2017-02-22

**Authors:** Richard Tushemereirwe, Doreen Tuhebwe, Mary Ann Cooper, Florence Mutonyi D'ujanga

**Affiliations:** African Centres for Lightning and Electromagnetics, Kampala, Uganda; Department of Health Policy, Planning & Management, School of Public Health, College of Health Sciences, Makerere University, Kampala, Uganda; African Centres for Lightning and Electromagnetics Network, Kampala, Uganda; Department of Physics, Makerere University, Kampala, Uganda

## Abstract

**Introduction**: It is estimated that five thousand people die on Lake Victoria every year by drowning which is triggered by severe weather hazards like lightning.

**Objectives**:  In order to improve predictability of severe weather conditions on Lake Victoria, there is need to deliver timely and effective Severe Weather Early Warning Systems (SWEWS) to those at risk. On Lake Victoria, previous SWEW service trials ceased with the end of the funding grants. This study therefore assessed the possibility of sustaining the SWEW service by assessing willingness to pay.

**Methods**: An assessment was conducted between March and May 2015 to determine the SWEW service improvements desired by the population. A convenience sample of respondents was gathered and interviewed during impromptu visits to landing sites on Lake Victoria. The respondents were also among community members that had earlier participated in a pilot assessing the feasibility of mobile phones is delivering SWEW alerts.  Semi-structured questionnaires were administered to fishermen and fisher folks at the landing site to gather suggestions/strategies for (i) better design and implementation of SWEW service, (ii) use of smart phones, and (iii) their ability and willingness to pay for a SWEW service. Results were presented as frequencies.

**Results**: Two hundred fifteen respondents from fourteen landing sites (communities) were interviewed. Over 50% of the respondents (113/215) were aware about at least one community member who had been injured due to lightening on the lake in the past year. Ninety two percent (198/215) of the respondents reported using mobile phones as their main tool of communication but only 4% had smart phones that could receive early warning weather alerts through internet connectivity. Seventy five percent of respondents said they would welcome a system that could deliver commercial weather alerts and 65% were willing to pay for such a service.

**Conclusions**: A SWEW service is feasible in this community but must be accompanied with public education on risk, a design that can fit the basic phone functionality and a system that the community majority will be willing to pay for on a continuing basis as a sustainability plan/strategy for an early warning system. This will enable timely dissemination of severe weather alerts and reduce risk of drowning on lakes among fishing communities.

## INTRODUCTION

According to the recent study, an estimated 5,000 people per year die from severe weather on Lake Victoria.^1^ The northern part of Lake Victoria experiences the maximum number of thunderstorms which average to 242 days per year compared to any other country in the world. Fishermen are at special risk of drowning, not only from high winds, waves and lightning but from poorly maintained boats, lack of life saving equipment and navigational aids. In addition, gale size winds can move inland to destroy vegetation, property and human settlements including schools and health centers.^2^

The loss of life impacts more than fishermen by affecting their families and communities both economically and socially. Persons who feel they have little control over their lives may become resigned to their fate. They may be less likely to listen to warnings of other risks such as HIV and more likely to choose life’s pleasures rather than pursue education for their children and other self-improvement activities that require time, energy, money, and sufficient hope that delays gratification becomes a viable behavior. The communities surrounding Lake Victoria are among those with the highest levels of HIV infection, poverty, disease, gender-based marginalization, lack of education, and violence.^3^

By providing weather information and warnings to fishermen, they are able to make informed decisions about when and where to fish, whether to go out onto the lake or seek shelter in safe areas. This not only helps save many lives, but also enhances the livelihoods of the communities around the lake, as many fishermen are the sole providers for large families.

In order to decrease the loss of lives, we propose to design a Severe Weather Early Warning System (SWEWS) utilizing lightning detection and other weather data in cooperation with the Uganda National Meteorological Administration, Earth Networks, and other agencies to deliver SWEWS by smart phone technology to fishermen and others who work around the Lake.

Previous SWEWS have been piloted in the communities living on Lake Victoria by the World Meteorological Organization (WMO) and Grameen Foundation in 2011-2012 but tended to fail when grant funds and personnel left. Most of these projects are no longer operating for a number of reasons, including problems with delivery to the end users, timeliness of information, and lack of sustainability given that the community was not paying a fee for this service. Other projects utilizing different segments of this idea have been implemented in the region including one for farmers to search for advice on managing crops and livestock, for weather data and market prices^4^ for fishermen

In summary, we propose a SWEWS with accurate and timely weather reports delivered to mobile phones in languages appropriate to the user. Many components are involved:1)Accurate weather data collection; 2) Skilled interpretation to provide reliable forecasts and timely warning; 3) Delivery of warnings in a timely and effective manner to the end-user; 4) Education on how to interpret the information delivered; 5) Implementation of alternative livelihood 6) Assurance that the SWEWS is economically sustainable

Before any of these can be effective, it is essential to determine what the end-users, particularly the fishermen and those living around the Lake, identify as important to its effectiveness as well as their willingness to support the system with subscriptions once it is implemented as part of the community sustainability strategy.

## METHODOLOGY

This study focused on the fishing community of Lake Victoria in Kalangala District, a major group at risk from severe weather effects. Kalangala District is located on the northern shore of Bugala Island, the largest of the Ssese Islands in Lake Victoria. Kalangala is made up of 84 widely scattered islands. It is approximately 60 kilometers across water, southwest of Entebbe, the largest and nearest mainland town. In 2011, the Uganda Bureau of Statistics (UBOS) estimated the mid-year population of Kalangala district at 5,200. People settle mostly in villages made of temporary structures with each village having approximately 200 households. The principal economic activity on the Island is fishing. Nile Perch is the species primarily fished with most of the catch processed on the mainland for export. Other economic activities include agriculture, crop and animal husbandry, logging and tourism.

Twenty four villages spread across five islands of Bubeke, Bukasa, Kachanga, Misozi and Bulagain in Kalangala District provided a convenience sample gathered during impromptu visits to interview those found on site. A total of 215respondents were sampled from the 1000 participants who had participated in the earlier pilot of Mobile Weather Alerts supported by World Meteorological Organization that ended in early 2011. They were specifically solicited, when possible, because their experiences were deemed valuable in planning an improved approach and in enhancing use by the target population.

The information collected was aimed at determining the elements of a people-centered EWS, i.e.

1) risk knowledge , 2) monitoring and warning service , 3) dissemination and communication and 4) response capability.

Written informed consent was sought from each individual before the questionnaire was administered. The study approval was sought from the Uganda National Meteorological Authority because this assessment was part of a follow up of the work done by the World Meteorological Organization to inform adjustments in the intervention. We did not obtain approval from an Institutional Review Board but rather approval from the Uganda national Meteorological Authority. The assessment was conducted under supervision of Makerere University School of Public Health

Respondents were assessed for; socio-demographic characteristics, experience with the Mobile Weather Alert pilot, extent of use of the mobile phone as an information source, knowledge and perceptions about lightning and severe weather including the features they desired to be part of a mobile weather alert system. Quantitative data was collected using a pretested semi-structured questionnaire which was developed based on the information gathered from previous pilots and included questions on; personal history, exposure to the Lake, perceptions about SWEWS, as well as, willingness and ability to pay for such a system or service.

The questionnaire was administered to the adults with the aid of a local language (Luganda) translator by trained research assistants utilizing iPads/tablet computers for data entry. Data was summarized using Microsoft Excel spreadsheets to obtain frequencies in each category of questions and data was presented by appropriate tables, figures and text.

## RESULTS

A total of 215 respondents from twenty four landing site communities/ villages were interviewed as summarized in Table 1. The table also highlights their socio- demographic characteristics. Most of the respondents were men (84%) and from Bulaga village. Over a half (52%) had attained primary education and 48% reported that their main occupation was fishing in the lake.

**Figure:**
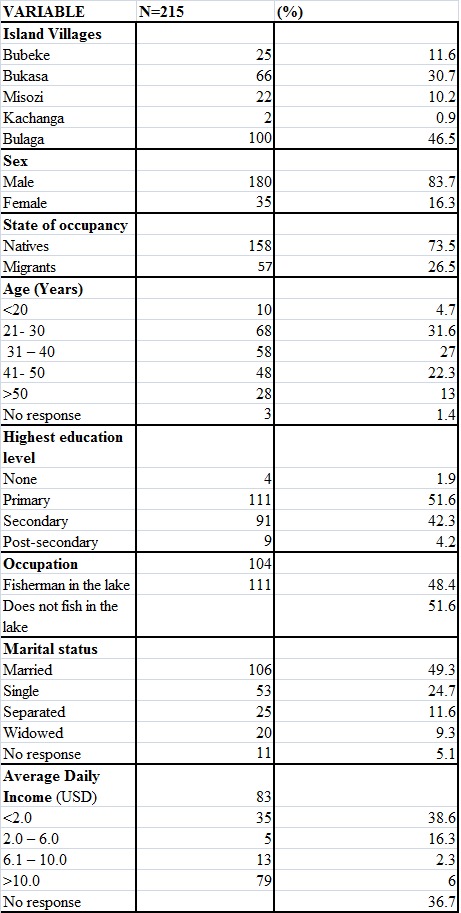
Table 1 - Demographics of Study Population

**Community exposure to the Lake**

In the study population, 27.9% (60/ 215) of the respondents reported that they owned their own boats. Boats were nearly equally split between those with motors versus those without (48.1 vs 51.9%). Over half of the respondents reported that they traveled by boat on Lake Victoria on a daily basis. (Fig 1),

**Figure:**
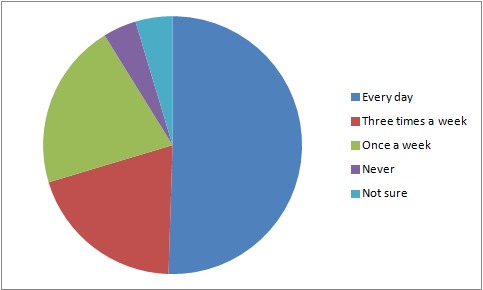
Fig. 1 - Frequency of travel by boat on the Lake

When asked about the community’s perception of severe weather effects, the majority of respondents (91.7%) cited a combination of different hazards, mainly high winds and thunderstorms. The same group noted that high winds come with high waves, while thunderstorms come with lightning, making it difficult to quantify each of these hazards separately (Table 2). Lightning injury alone was cited by only 6.5% as a major hazard. However, up to 54% of respondents could recall lightning strikes on a regular basis (24% everyday; 17% every week; and 13% every month).

**Figure:**
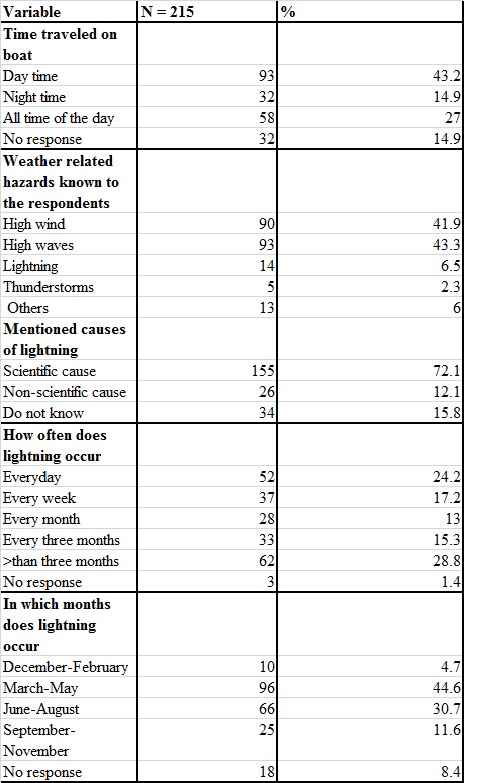
Table 2 - Perceptions of the community in relation to severe weather

Over 50% of the respondents (113/215) were aware about at least one community member who had been injured due to lightning on the lake in the past year. It was also noted that 32.6% (70/215) of the respondents knew at least one community member who had died on the lake due to lightning (Table 3). When asked specifically what they believed was responsible for these deaths, multiple responses were given including high winds (88%; 62/70) and 30% (21/70) cited poorly maintained boats.

**Figure:**
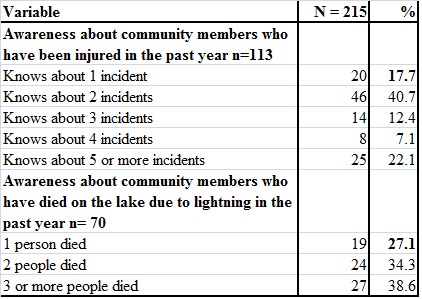
Table 3 - Knowledge about Injuries and Fatalities from Lightning on the Lake in the past year

**Level and extent of mobile phone use in the fishing community**

All respondents reported using mobile phones. Ninety-two percent (198/215) mentioned the mobile phone as their most useful tool for obtaining information, while 8 % mentioned the radio. The majority of the respondents 85.1% (183/215) paid for their own mobile usage fees. On average, the respondents reported that they spend USD 8 per month for mobile phone services. In the study population, 96% of the respondents (206/215) had mobile phones with basic functionality and only 4% (9/215) had advanced features.

**Use of mobile alert systems**

It was reported that 30.7% (66/215) of the respondents were currently receiving mobile phone weather alerts. When these respondents were asked how the mobile phone alerts were helping them, 80.3% (53/66) said that the alerts were helping them in planning while 2.8% (6/66) said that the alerts informed them about the weather conditions. However 70.2% (151/215) of the respondents were not receiving any mobile phone weather alerts. Seventy-five percent of respondents said they would welcome a system that could deliver commercial weather alerts, while 65% were willing to pay for such a service. Table 4 also illustrates how respondents thought that alerts on mobile phones could be made more effective.

**Figure:**
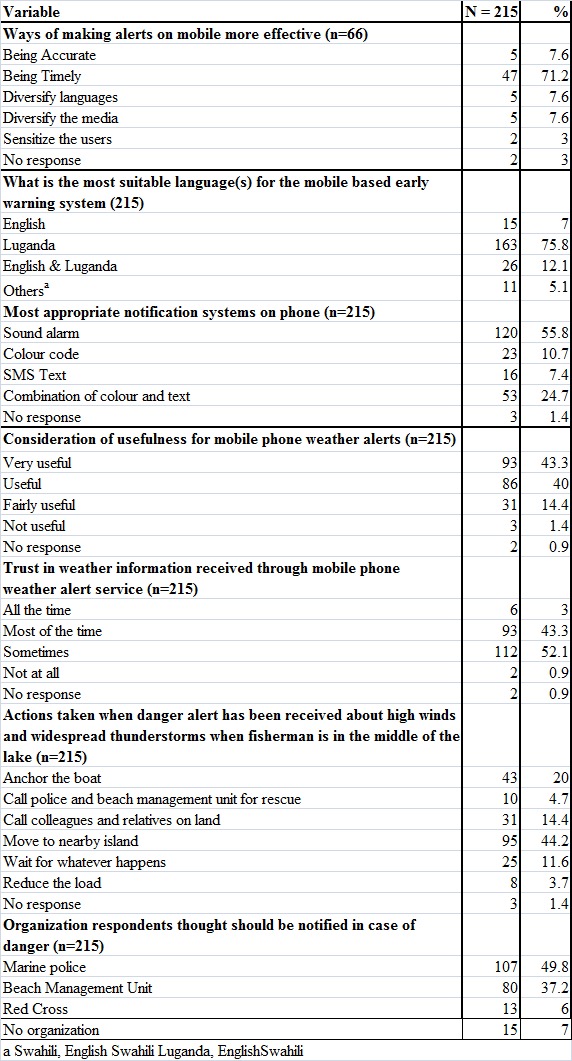
Table 4 – Preferred Characteristics of Severe Early Weather Warnings

**DISCUSSION**

The populations that depend on fishing for their livelihoods is of high economic importance in Uganda's economy since the fishing industry contributes 2.48% to the Uganda’s GDP.^5^ The fact that so many respondents could recall more than two incidences with three or more deaths within the past year speaks to the loss of life and to the many productive years. Such loss to the economy and family support due to premature death alludes to the need for risk reduction through early warning.^6^ In addition, the fact that over half of those studied travel on boats every day points out the risk are exposed to.

The aim of this study was to gather information that would be used to improve the delivery of potentially life-saving weather warnings. These warnings would enable fishermen and others in the community to make informed decisions about their work and exposure to severe weather. For that reason, it was good news to note that most of the respondents in the sampled villages were natives, making it easier to build a trusted and sustainable business model for the early warning delivery. It is much more difficult to build a sustainable enterprise with migrant workers and their dependent populations.^7^ In order to assure sustainability in building EWS where community members pay for the system or service through subscriptions, it is important to choose a warning communication technology that is acceptable to the recipients. Among other considerations is the; recipient location, their activity, the systems they rely on to receive local news and information, any special needs they may have and how they understand and respond to warnings.^8^

In this study, all respondents owned mobile phones, relied on them as their most common source of information, and spent 8 USD per month on average for airtime credit. This presents the opportunity to harness the mobile phone alerts to their willingness and ability to pay for severe weather alerts in order to support a sustainable EWS. However, for this particular project to succeed, it will be necessary to consider the functionality of the phone and to either fit the EWS to basic phones or to make smart phones available at a low cost.

The study revealed some parts of the population at risk during all hours of the day and night as they routinely traveled and worked on the Lake. This support the need for an effective EWS that incorporates; risk knowledge, appropriate monitoring of the warning service, timely dissemination to the populations at risk and enhanced response capacity of the community.^8^^,^^9^ As far as risk knowledge, the majority of respondents were aware of severe weather related hazards on the lake and believed a scientific explanation for lightning, rather than a superstitious or supernatural explanation for its source. This implied that they will see scientific methods as reliable for giving them warning and improving their control over lightning risk. The proposed severe weather casting early warning system aims to use lightning as a proxy measure of severe weather, a sound and scientific basis for early warning on the lake that regularly provides hazards like heavy winds, waves and thunderstorms.^10^

Concerning dissemination and communication, respondents reported that they rely heavily on mobile phones for information. The few people who had experience with mobile phone weather alerts in earlier projects had found the alerts useful especially in planning, showing that warnings reached the intended users, were understood and usable.

Most respondents preferred an alarm as a warning system over color codes or text. This may be due to the low literacy levels among the fisher folk which could impede reading text warnings to ensure timely response. They also agreed that timeliness was the most important factor and thus preferred the use of local language for EWS.

In this study, the community noted their trust in the SWEWS that had been piloted earlier which speaks to the perceived validity of the warning messages. In addition, authorities like the police were perceived as the responsible body that should issue the alerts. This is in agreement with the incident command operations that call for a clear chain of information flow.

The most common response action to a severe weather alert reported in this community was moving to a nearby island for safety and anchoring the boat. Although validation of this response was not tested as part of this study. The use of such indigenous/ local knowledge, if effective, may improve the effectiveness of response to an early warning system. As part of the warning dissemination, such information should be widely available so that there is organized response capacity in the community

There are some limitations to this study worth mentioning. Given that a convenience sample of individuals was taken, the results may be biased in a way that they don’t represent the views of the general population. In addition, some communities had few respondents compared to others. Nevertheless, we believe that the information can aid in the design of an acceptable tool for early warning with the best delivery mechanisms, given that we interviewed the population that is aware of the hazards and risks associated with the Lake and severe weather effects.

The study sample was not chosen randomly and this may have caused reporting bias. In addition, a portion of the population targeted had participated in the pilot study of testing a mobile phone weather alert service. However, since the pilot interventions had ended over a year ago, we believe that the experience of the respondents and their responses may actually enhance the next system and that the results of this assessment are valid enough to guide an improved approach. This will enable timely dissemination of severe weather alerts against hazards like lightning and thunderstorms hence giving lead time to those exposed to take actions to prevent, mitigate or prepare for such risks. The severe weather early warning system will hence reduce risk of drowning on lakes among fishing communities.

**CONCLUSION**

Exposure to the lake was common with fishing in the lake being a major occupation in the study population. Over half of the respondents were aware of at least one community member who had been injured due to lightning and a significant number of respondents were willing to pay for a smart phone based commercial severe weather early warning system service. This will enable timely dissemination of sever weather alerts against hazards like lightning and thunderstorms hence giving lead time to those exposed to take actions to prevent, mitigate, or prepare for such risks. The sever weather early warning system will therefore reduce risk of drowning on lakes among fishing communities.

## Corresponding Author

Doreen Tuhebwe (dtuhebwe@musph.ac.ug; tuhereen@yahoo.com).

## Competing Interests

The authors have declared that no competing interests exist.

## Data Availability Statement

All relevant data are within the manuscript and the public repository Figshare. Please view data at the following URL: https://figshare.com/s/526fec13e1c8e386809d

## Ethics Statement

Approval for the study was received from the Uganda National Meteorological Authority. The assessment was conducted under supervision of Makerere University School of Public Health.
